# TRK inhibitors in TRK fusion-positive cancers

**DOI:** 10.1093/annonc/mdz282

**Published:** 2019-11-18

**Authors:** A Drilon

**Affiliations:** 1 Memorial Sloan Kettering Cancer Center, New York; 2 Weill Cornell Medical College, New York, USA

**Keywords:** TRK, tropomyosin receptor kinase, *NTRK* gene fusions, TRK fusion cancer

## Abstract

TRK fusions are oncogenic drivers of various adult and paediatric cancers. The first-generation TRK inhibitors, larotrectinib and entrectinib, were granted landmark, tumour-agnostic regulatory approvals for the treatment of these cancers in 2018 and 2019, respectively. Brisk and durable responses are achieved with these drugs in patients, including those with locally advanced or metastatic disease. In addition, intracranial activity has been observed with both agents in TRK fusion-positive solid tumours with brain metastases and primary brain tumours. While resistance to first-generation TRK inhibition can eventually occur, next-generation agents such as selitrectinib (BAY 2731954, LOXO-195) and repotrectinib were designed to address on-target resistance, which is mediated by emergent kinase domain mutations, such as those that result in substitutions at solvent front or gatekeeper residues. These next-generation drugs are currently available in the clinic and proof-of-concept responses have been reported. This underscores the utility of sequential TRK inhibitor use in select patients, a paradigm that parallels the use of targeted therapies in other oncogenic driver-positive cancers, such as ALK fusion-positive lung cancers. While TRK inhibitors have a favourable overall safety profile, select on-target adverse events, including weight gain, dizziness/ataxia and paraesthesias, are occasionally observed and should be monitored in the clinic. These side-effects are likely consequences of the inhibition of the TRK pathway that is involved in the development and maintenance of the nervous system.

**Key Message**

Larotrectinib and entrectinib are first-generation TRK inhibitors and have demonstrated rapid and durable responses and favourable safety profiles in patients with TRK fusion-positive cancers. Intracranial activity has also been observed with both larotrectinib and entrectinib in patients with TRK fusion-positive solid tumours with brain metastases or primary brain tumours.

## Introduction

Cancer can be driven by a wide variety of clinically actionable alterations. The late 1990s and 2000s were marked by landmark regulatory approvals of targeted therapies that exploit these dependencies. These included the US Food and Drug Administration (FDA) approval of trastuzumab for HER2-positive breast cancers in 1998, imatinib for Philadelphia chromosome-positive chronic myelogenous leukaemias in 2001, and gefitinib for *EGFR*-mutant lung cancers in 2009 [1]. These sparked the subsequent approval of a growing list of matched therapeutics for activating alterations involving *EGFR, BRAF, ALK* and *ROS1* by a wider umbrella of one or more additional regulatory agencies in Europe, Asia and/or Latin America*.* While these approvals have undoubtedly reshaped the therapeutic landscape for patients with a wide array of cancers, the respective drug development programmes largely focussed on establishing the activity of these agents within a single tumour type.

An increase in the adoption of more comprehensive, clinical-grade sequencing platforms later promoted a recognition of the presence of targetable genomic alterations across multiple tumour types [2]. Consequently, select drug development programmes migrated away from histology-specific patient selection to biomarker-driven, tumour-agnostic enrichment. This gave birth to the basket trial strategy under which patients were accrued to a trial of a matched therapeutic regardless of histology as long as their cancers harboured the appropriate genomic signature [3]. Elucidation of the activity of pembrolizumab in tumours of any type with high levels of microsatellite instability (MSI-high) marked the first major success in this area [4]. This culminated in the US FDA approval of pembrolizumab for this indication in 2017.

The same year pembrolizumab was approved for MSI-high cancers of any type, the first-generation TRK inhibitors larotrectinib and entrectinib were granted breakthrough designation by the US FDA for the treatment of TRK fusion-positive cancers of any histology. Shortly thereafter, in 2018, larotrectinib received accelerated approval by the FDA for the treatment of TRK fusion-positive cancers of any type and any age.* This was followed in 2019 by the approval of entrectinib for TRK fusion-positive cancers in Japan and the US. Remarkably, the activity of next-generation TRK inhibitors, such as selitrectinib (BAY 2731954, LOXO-195) and repotrectinib, is already being explored in ongoing clinical trials.

## First-generation TRK inhibitors

### Preclinical activity

Larotrectinib and entrectinib are first-generation TRK tyrosine kinase inhibitors with potent activity against wild-type TRKA, TRKB and TRKC (Table 1). In enzymatic assays, larotrectinib and entrectinib have IC_50_ values of 5–11 nM and 1–5 nM, respectively, for TRKA/B/C [5, 6]. However, the drugs differ in their activity against other kinases. Larotrectinib is a selective inhibitor of TRKA/B/C. It has greater than 100-fold selectivity against 229 other kinases and greater than 1000-fold selectivity against 80 non-kinase targets [7]. By contrast, entrectinib is a multikinase inhibitor. In addition to TRKA/B/C, it inhibits ROS1 (enzymatic IC_50_ of 0.2 nM) and ALK (enzymatic IC_50_ of 1.6 nM) [8, 9].

**Table 1.** TRK inhibitors

	Larotrectinib	Entrectinib	Selitrectinib	Repotrectinib
Generation				
First	✓	✓		
Second			✓	✓
Inhibits				
TRKA/B/C	✓	✓	✓	✓
ROS1		✓		✓
ALK		✓		✓
Resistance				
Inhibits most *NTRK* mutations			✓	✓

The features of four TRK tyrosine kinase inhibitors (larotrectinib, entrectinib, selitrectinib and repotrectinib) are summarised by tyrosine kinase inhibitor generation, major kinase targets and activity against resistance.

Larotrectinib and entrectinib are active against *in vitro* and *in vivo* models harbouring TRK fusions [9–11]. Larotrectinib effectively inhibits the growth of cell lines or xenografts containing *TPM3-NTRK1*, *MPRIP-NTRK1*, *TRIM24-NTRK2* or *ETV6-NTRK3*. This is associated with downstream inhibition of the RAF-MEK-ERK or PI3K-AKT pathways [11]. Similarly, entrectinib inhibits the growth of cell lines or xenografts containing *LMNA-NTRK1* or *EVT6-NTRK3*, with the consequent inhibition of downstream pathway signalling [9].

Other multikinase agents have varying degrees of activity against TRKA/B/C. These include cabozantinib, crizotinib, nintedanib and ponatinib, which have regulatory approval for other indications, and the investigational agents altiratinib, foretinib, lestaurtinib, merestinib, MGCD516, PLX7486, DS-6051b and TSR-011 [12]. It is important to note that several of these drugs are less potent against TRK (IC_50_s of 50 to >200 nM for nintedanib and ponatinib [12, 13]) compared with larotrectinib or entrectinib. Furthermore, while the preclinical activity of some of these drugs against TRK fusion-containing models has been described, their clinical activity has not been as well characterised as that of larotrectinib and entrectinib.

### Drug development programmes

The two major drug development programmes for larotrectinib and entrectinib share several features. The regulatory data sets for both agents included patients with TRK fusion-positive cancers who were treated on several clinical trials. For larotrectinib, the three trials that contributed patients were an adult phase I trial, a paediatric phase I/II trial (SCOUT) and an adult/adolescent phase II basket trial (NAVIGATE), all of which enrolled patients with advanced solid tumours [5]. For entrectinib, the four contributory trials were an adult phase I trial (ALKA-372-001, Italy), a separate adult phase I trial (STARTRK-1, global), a phase II basket trial (STARTRK-2), which enrolled patients with solid tumours harbouring an *NTRK*1/2/3, *ROS1* or *ALK* gene fusion, and a phase I/Ib paediatric trial (STARTRK-NG) [8, 14].

The activity of these agents was analysed in both data sets in aggregate, with the primary enrichment factor being the presence of an *NTRK* gene fusion regardless of cancer type [5, 8]. A wide variety of tumour types were treated with either agent. These included the four histologies enriched for the presence of an *NTRK* gene fusion (mammary analogue secretory carcinoma, secretory breast carcinoma, infantile fibrosarcoma and congenital mesoblastic nephroma), and several other malignancies including lung, gastrointestinal, breast and thyroid cancers, melanoma and soft tissue sarcoma. Objective response was the primary end point of both programmes [5, 8].

### Clinical activity

In 2018, the antitumour activity of larotrectinib in the first 55 adult and paediatric patients consecutively enrolled into one of the three larotrectinib trials was published [5]. The objective response rate (ORR) was 75% [95% confidence interval (CI) 61% to 85%, independent review]. In the 15 paediatric patients with evaluable disease in this series, the ORR was 93% (95% CI 68% to 100%) [15]. This initial data set has since been expanded [16]. A total of 122 adult and paediatric patients (including the 55 patients above) with TRK fusion-positive cancers have since been treated with larotrectinib. Age ranged from 1 month to 80 years. The most common histologies were salivary gland cancer (16%), infantile fibrosarcoma (15%), thyroid cancer (15%) and lung cancer (9%). Most fusions involved *NTRK1* (45%) or *NTRK3* (53%) and were detected by local molecular profiling [5].

In this updated data set, the ORR was 81% (95% CI 72% to 88%; *n *=* *109 evaluable; Table 2). Response occurred regardless of tumour type, age, *NTRK* gene or upstream partner type. Complete and partial responses were observed in 17% and 63% of patients, respectively. The median duration of response was not reached. The median time to response was 1.8 months (approximately when the first follow-up imaging assessment was carried out). The median progression-free survival (PFS) and overall survival (OS) have yet to be reported for the larger integrated set [16]; neither had been reached at the earlier data cut in the first 55 patients [5].

**Table 2.** TRK inhibitor activity

	Larotrectinib (*n* = 122)	Entrectinib (*n* = 54)
ORR (95% CI)	81% (72% to 88%)	58% (43% to 71%)
CR	17%	–
PR	63%	–
Median DoR, months	Not reached	10.4
Median PFS, months	Not reached	11.2
Median OS, months	Not reached	20.9

The clinical activity of the first-generation TRK inhibitors, larotrectinib and entrectinib, is summarised. Data for the breakdown of CRs/PRs with entrectinib not available.

CI, confidence interval; CR, complete response; DoR, duration of response; ORR, objective response rate; OS, overall survival; PFS, progression-free survival; PR, partial response.

Data from this drug development programme resulted in the approval of larotrectinib by the US FDA for the treatment of TRK fusion-positive cancers regardless of tumour type or age in November of 2018. This label includes a provision for the treatment of cancers that are locally advanced based on the experience with neoadjuvant therapy in this programme. The most illustrative cases involved infants with infantile fibrosarcomas who would have required limb amputations to treat their cancer. Larotrectinib use resulted in substantial disease regression (complete pathological response) that allowed limb-sparing surgery, underscoring the utility of this therapy in earlier stage disease [15].

A total of 54 patients with TRK fusion-positive cancers were treated with entrectinib [17]. Notably, this initial data set only included adult patients with a median age of 58 years (range 21–83 years). The most common histologies were sarcoma (24%), lung cancer (19%), mammary analogue secretory carcinoma (13%) and breast cancer (11%). The majority of fusions involved *NTRK1* (41%) or *NTRK3* (57%). The ORR was 57% (95% CI 43% to 71%; *n *=* *54 evaluable; Table 2) [17]. Response occurred regardless of tumour type. Response did not differ between fusions involving *NTRK1* or *NTRK3*. The median duration of response was 10.4 months [17]. The median PFS and OS were 11.2 and 20.9 months, respectively [17].

The activity of entrectinib in paediatric patients on the STARTRK-NG trial was thereafter presented [14]. Seven patients with TRK fusion-positive cancers were treated. These cancers were high-grade gliomas (*n* = 3), a central nervous system (CNS) embryonal tumour (*n *=* *1), melanoma (*n *=* *1) and infantile fibrosarcomas (*n *=* *2). Most fusions involved *NTRK3* (*n* = 4). All six patients with measurable disease had an objective response to therapy with >50% target tumour shrinkage; disease control was durable with the longest duration of ongoing benefit lasting close to 15 months.

Based on data from this drug development programme, entrectinib was granted Breakthrough Designation by the US FDA for the treatment of TRK fusion-positive cancers in May of 2017. This was followed in June of 2019 by the approval of entrectinib in Japan by the Ministry of Health, Labour and Welfare, and in August of 2019 by the US FDA for the treatment of adult and paediatric patients (age 12 years and above, US FDA) with advanced or recurrent TRK fusion-positive cancers. These were likewise landmark events, particularly the Japanese approval that represented the first tumour-agnostic approval of a targeted therapy for a specific genomic signature in Asia.

### Intracranial activity

*NTRK* gene fusions are identified in primary brain tumours and extracranial solid tumours with a proclivity for brain metastases (e.g. lung cancers) [12]. The activity of TRK inhibitors in the CNS is thus highly relevant. Fortunately, intracranial disease control has been achieved with both entrectinib and larotrectinib in patients with TRK fusion-positive primary brain tumours and brain metastases [14, 18–21].

The adult entrectinib data set was unique in that a higher proportion of patients (22%; *n *=* *12/54) had baseline CNS metastases relative to the larotrectinib data set [17]. The intracranial ORR with entrectinib was 55% (95% CI 23% to 83%). Complete and partial intracranial responses were each observed in 27% (*n *=* *3) of patients. The median intracranial duration of response was not reached. The median intracranial PFS was 14.3 months. In paediatric patients, all three TRK fusion-positive high-grade paediatric gliomas had durable responses to entrectinib on the STARTRK-NG trial [14].

In the larotrectinib programme, only 5% of adult and paediatric patients with TRK fusion-positive solid tumours (*n *=* *6/122) had baseline CNS metastases [20]. Among these patients (two thyroid cancers, four non-small-cell lung cancers), the ORR was 60% (*n *=* *3/5 evaluable). Intracranial disease regression was achieved in all three patients with measurable CNS disease; intracranial disease control was achieved in patients with evaluable but non-measurable CNS disease [20, 21]. Eighteen patients with TRK fusion-positive primary CNS tumours were treated with larotrectinib. The ORR was 36% (*n *=* *5/14 evaluable; two complete responses and three partial responses) and no primary disease progression was observed. The median PFS was 11 months [20].

## TRK inhibitor resistance

### On-target resistance

TRK fusion-positive cancers can develop resistance to TRK inhibition despite continued reliance on TRK fusion signalling [12]. These on-target resistance mechanisms take the form of *NTRK* kinase domain mutations. Interestingly, these mutations are paralogous to resistance mutations that have been identified after progression on ALK tyrosine kinase inhibitor therapy in ALK fusion-positive lung cancers, and ROS1 tyrosine kinase inhibitor therapy in ROS1 fusion-positive lung cancers [22, 23].

These kinase domain mutations result in amino acid substitutions that involve three major regions: the solvent front, the gatekeeper residue or the xDFG motif. Mutations in the *NTRK* kinase domain cause resistance to TRK inhibitors by sterically interfering with binding of the inhibitor, altering the kinase domain conformation or altering ATP-binding affinity [24, 25]. Solvent front substitutions include TRKA^G595R^, TRKB^G639R^ and TRKC^G623R^ [12] that are paralogous to ALK^G1202R^ and ROS1^G2032R^. Gatekeeper substitutions include TRKA^F589L^, TRKB^F633L^ and TRKC^F617L^ that are paralogous to ALK^L1196M^ and ROS1^L2026M^ [26]. xDFG substitutions include TRKA^G667C^, TRKB^G709C^ and TRKC^G696A^ that are paralogous to ALK^G1269^ substitutions.

Several of these mutations have been identified in patients with TRK fusion-positive cancers that have progressed on entrectinib [10, 24] or larotrectinib [20], indicating that these represent a shared liability for the first-generation TRK inhibitors. The majority of these clinically identified kinase domain mutations unsurprisingly involve *NTRK1* or *NTRK3*, given that few patients with *NTRK2* gene fusions have been treated with a TRK inhibitor [5, 17]. The true frequency of on-target resistance in TRK fusion-positive cancers has yet to be determined. However, the identification of these alterations in multiple patients implies that these events are unlikely to be rare.

### Off-target resistance

Similar to ALK and ROS1 fusion-positive lung cancers [26], TRK fusion-positive cancers can develop off-target resistance to tyrosine kinase inhibitor therapy. These mechanisms take the form of genomic alterations involving other receptor tyrosine kinases or downstream pathway mediators. Specifically, *MET* amplification, *BRAF*^V600E^ mutation or hotspot mutations involving *KRAS* have been shown to emerge in tumour and/or plasma samples from patients with TRK fusion-positive cancers that have progressed on a TRK inhibitor [27]. These alterations were likewise identified in preclinical models of TRK inhibitor resistance. IGF1R activation is also thought to play a potential role in mediating resistance [13]. The relative frequency of off-target resistance has yet to be determined.

Combination therapy has been shown to re-establish disease control in the face of off-target resistance. For example, the combination of a TRK and MET inhibitor achieved a confirmed response to therapy in a patient with a TRK fusion-positive cancer with *MET* amplification-driven resistance to a first-generation TRK inhibitor [27].

## Next-generation TRK inhibitors

### Preclinical activity

Next-generation TRK inhibitors were specifically designed to address on-target resistance mutations while maintaining potency against wild-type TRKA/B/C (Table 1). The two major agents that are currently in development are selitrectinib and repotrectinib. Due to their small size, these low molecular weight macrocycles are able to engage the ATP-binding pocket while avoiding the steric penalties of kinase domain substitutions [25, 28].

Both drugs have increased activity against wild-type TRKA/B/C compared with the first-generation TRK inhibitors. For solvent front substitutions, the IC_50_s of selitrectinib and repotrectinib in enzymatic assays are 2.0–2.3 nM and 2.7–4.5 nM, respectively. For gatekeeper substitutions, the IC_50_s of selitrectinib and repotrectinib are 2.0–2.3 nM and <0.2 nM, respectively. For xDFG substitutions, the IC_50_s of selitrectinib and repotrectinib are 2.0–2.3 nM and 9.2 nM, respectively. This activity is echoed in select *in vitro* and *in vivo* models, particularly for those that contain solvent front and gatekeeper substitutions [25, 28, 29].

### Clinical activity

The clinical activity of selitrectinib (NCT03215511) and repotrectinib (NCT03093116) are currently being explored in separate phase I/II trials. In addition, patients have been treated with selitrectinib [25] under expanded access/compassionate use protocols (NCT03206931). The largest data set reported thus far has been the experience with selitrectinib [30].

In total, 31 patients with TRK fusion-positive cancers received selitrectinib [30]. All patients were treated with a prior TRK inhibitor (larotrectinib, entrectinib or PLX7486) with a median duration of prior tyrosine kinase inhibitor therapy of 11 months (range 2–30 months). The most common histologies were sarcoma (16%), gastrointestinal stromal tumour (13%) and pancreatic adenocarcinoma, mammary analogue secretory and breast carcinoma (10% each). In patients with TRK kinase domain mutations (the majority of which involved the solvent front), the ORR was 45% (*n *=* *9/20). None of the three patients with identified bypass mechanisms of resistance responded to therapy. In addition, none of the five patients with unknown mechanisms of resistance (excluding one additional patient with prior TRK inhibitor intolerance) responded to therapy.

Similarly, repotrectinib has been shown to re-establish disease control after the development of kinase domain-mediated acquired resistance to a first-generation TRK inhibitor [28]. A confirmed partial response was achieved in a patient with TRK fusion-positive mammary analogue secretory carcinoma and TRKA^G623E^-mediated resistance to entrectinib [28]. In addition, disease regression was observed in a patient with a TRK fusion-positive cholangiocarcinoma with TRKA^G595R^- and TRKA^F589L^-mediated resistance to larotrectinib [29].

**Figure 1.**
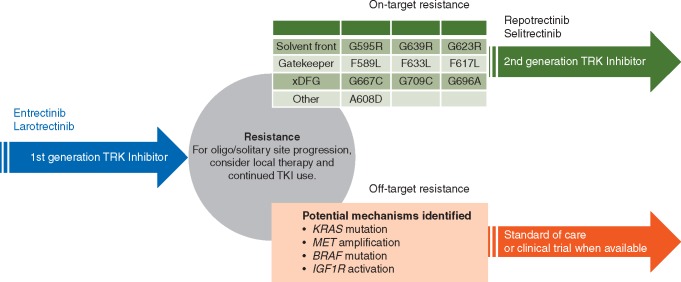
Sequential therapy. Durable responses to first-generation TRK inhibitors can be achieved in TRK fusion-positive cancers. In patients with advanced disease, when solitary site progression or oligoprogression occurs, local therapy such as radiation or surgery should be considered. The use of local therapy and continued treatment beyond progression has been shown to prolong disease control with TRK inhibitor therapy. At the onset of resistance, tumours can acquire either on-target or off-target resistance mechanisms. In the face of widespread disease progression, a second-generation TRK inhibitor trial could be considered for tumours that harbour on-target resistance, as evidence by the acquisition of solvent front, gatekeeper or xDFG TRKA/B/C substitutions. If a cancer has developed clear off-target resistance, disease-specific standard of care therapies could be considered if a clinical trial that meaningfully addresses these resistance mechanisms is not available. TKI, tyrosine kinase inhibitor.

This clinical activity highlights an evolving strategy for the treatment of TRK fusion-positive cancers (Figure 1). Next-generation TRK inhibitors can salvage resistance to a first-generation TRK inhibitor in select cases, a paradigm similar to that observed for ALK or ROS1 fusion-positive lung cancers [26]. The responses noted thus far have been in patients whose cancers clearly harbour on-target mechanisms of resistance [30], encouraging repeat molecular profiling of tumour and/or plasma in order to identify these alterations at the onset of resistance.

## TRK inhibitor safety

### Overall safety profile

The safety of TRK inhibition has been best characterised for the first-generation agents larotrectinib and entrectinib. Each drug has been given to more than 200 patients (207 for larotrectinib and 355 for entrectinib). These safety data sets include patients who were treated with these drugs regardless of molecular profile or cancer type.

Both agents had favourable overall toxicity profiles [16, 17]. Compared with other tyrosine kinase inhibitors, the frequency of treatment-emergent adverse events (only reported thus far for larotrectinib) was low. For example, the most common treatment-emergent adverse event for larotrectinib (fatigue) was observed in 36% of patients. The frequency of drug-related adverse events (reported for both larotrectinib and entrectinib) was also low [16, 17].

**Figure 2.**
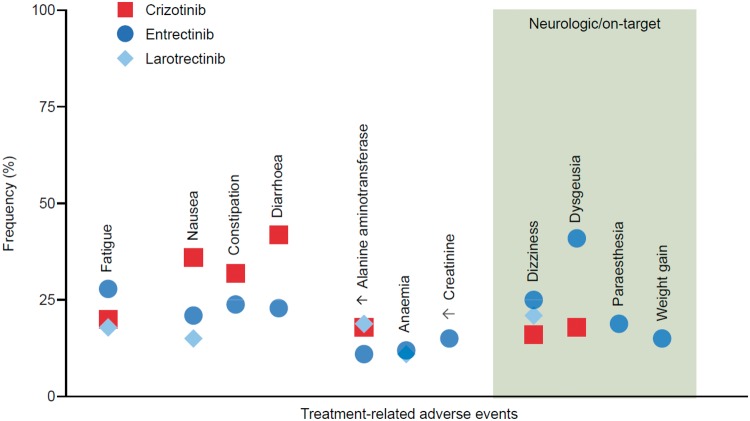
Safety profile. The frequency of select treatment-related adverse events with entrectinib and larotrectinib is shown. To benchmark the relative frequency of these toxicities, crizotinib was chosen for comparison as the drug has a well-known safety profile (and incidentally has minimal anti-TRK activity). These adverse events (present in 10% or more of patients) are grouped from left to right by the following categories: general (fatigue), gastrointestinal (nausea, constipation, diarrhoea) and laboratory abnormalities (increased alanine aminotransferase, anaemia, increased creatinine). The right-hand panel highlights adverse events that are predicted or presumed to be on-target neurologic consequences of TRK inhibition, recognising the importance of this pathway in neuronal development and maintenance.

By way of illustration, the frequencies of treatment-related nausea, diarrhoea and transaminitis were lower with entrectinib [17] or larotrectinib [16] than with crizotinib [31], a multikinase inhibitor with a well-characterised safety profile that incidentally has minimal activity against TRK (Figure 2). A notable side-effect unrelated to TRK inhibition that has been observed in the entrectinib data set was elevated creatinine. Importantly, this rise in creatinine is thought to be secondary to MATE1 transporter inhibition by the drug and may not be a true reflection of renal insufficiency [14].

**Figure 3.**
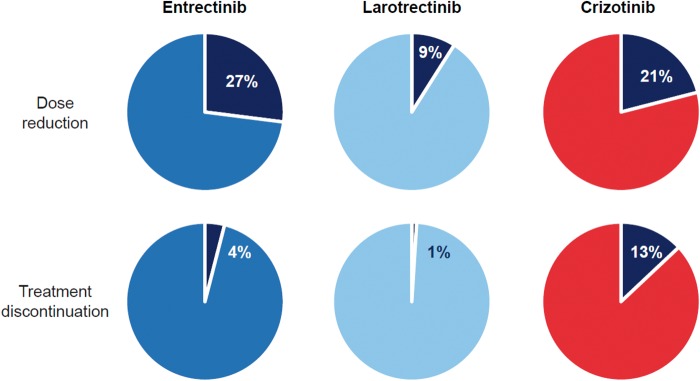
Dose modification. The rates of dose reduction and treatment discontinuation are shown for entrectinib and larotrectinib. To benchmark the relative frequency of these dose modifications, crizotinib was chosen for comparison as the drug has a well-known safety profile and incidentally has minimal anti-TRK activity.

The frequency of dose reduction and treatment discontinuation arguably represent the best integrated metrics for overall tolerability (Figure 3) [16, 17]. For comparison, the frequency of dose reduction with entrectinib [17], larotrectinib [16] and crizotinib [32] was 27%, 9% and 21%, respectively. The frequency of treatment discontinuation was consistently lower with both entrectinib (4%) and larotrectinib (<1%) than with crizotinib (13%).

### On-target adverse events

Occasional on-target adverse events can occur secondary to TRK inhibition in normal tissues [12]. These include dizziness, paraesthesia, weight gain and cognitive changes (Figure 2) [5, 8], predicted by the role that the TRK signalling pathway plays in nervous system development and maintenance [33]. Most of these adverse events are grade 1 or 2 in nature [5, 8], highlighting that while loss or inhibition of TRKA/B/C can have substantial consequences during embryonic development, the post-embryonic consequences of TRK inhibition can be relatively mild in comparison [12].

Dizziness occurs in approximately 16% to 25% of patients treated with larotrectinib or entrectinib [5, 8]. Based on early data in a small subset of patients, the frequency of dizziness/ataxia/gait disturbance rises with next-generation TRK inhibition and has been described as a dose-limiting toxicity of selitrectinib [30]. While clinical trials of first-generation TRK inhibitors have described this event as ‘dizziness’ for several patients, this side-effect can be attributed to decreased proprioception and cerebellar dysfunction. *Ntrk2*-knockout and *Ntrk3*-null mice lack dorsal root ganglia neurons [34, 35], and decreased levels of a TRK receptor ligand (BDNF) have been associated with cerebellar neuron pathology [36]. The description of ‘ataxia’ with next-generation TRK inhibition supports this hypothesis [30], and the more pronounced nature of this side-effect may be related to the more potent inhibition of wild-type TRK with these drugs (compared with first-generation TRK inhibitors).

Paraesthesias occur in a subset of patients (∼19% with entrectinib) [37]. *Ntrk1*-null mice suffer from severe sensory and sympathetic neuropathies [38] and loss-of-function *NTRK1* mutations are identified in paediatric patients with congenital insensitivity to pain and anhidrosis [39]. Despite this, all paraesthesias reported have been grade 1 or 2 in severity (with no grade 3 or greater events [Common Terminology Criteria for Adverse Events [40]]) [17].

Weight gain has also been observed. This occurs as TRKB is involved in the regulation of appetite [35] and impaired TRKB activity causes hyperphagia, obesity and hyperdipsia in mice and/or humans [41, 42]. In adults, the frequency of weight gain is ∼19% (treatment related) with entrectinib [17] and has not been reported as occurring in 15% or more of patients (treatment emergent) with larotrectinib [16]. Preliminary data show that the frequency might be higher in the paediatric population (28% [treatment related] with entrectinib in STARTK-NG where weight gain was the most common reason for dose reduction [and may have been associated with skeletal events such as fracture] [14] and 18% [treatment emergent] with larotrectinib [43]).

A dose-limiting toxicity of grade 3 cognitive disturbance was described with entrectinib in a patient treated at a dose above the recommended phase II dose of the drug [8]. This is consistent with the fact that the TRKB pathway plays a role in modulating memory and mood [44]. Cognitive changes have otherwise not been identified as a frequent side-effect at the recommended phase II doses of larotrectinib (100 mg twice daily) or entrectinib (600 mg daily).

Dysgeusia or a distortion in the sense of taste has been observed. This symptom can be caused by a variety of pathologies, among which neurological damage is a differential. While this is listed as a potential on-target adverse event in a select series [14], it is yet unclear that the TRK pathway plays a strong neurological role in the regulation of taste.

Finally, as TRK inhibitors were first developed as pain medications [45], these agents may modulate pain sensitivity, and patients who discontinue or hold these agents should be observed for potential pain flares. These could conceivably be managed by pain medication and by more slowly weaning patients off TRK inhibitor therapy, but data regarding the utility of the latter approach have yet to emerge.

## Summary

TRK fusions are actionable oncogenic drivers of paediatric and adult cancers. The treatment of patients with TRK fusion-positive cancers with a first-generation TRK inhibitor achieves high response rates irrespective of tumour histology, age or fusion type. In spite of durable disease control in many patients, some advanced TRK fusion-positive cancers eventually become refractory to TRK inhibition. Resistance can be mediated by the development of *NTRK* kinase domain mutations that can be overcome by next-generation TRK inhibitors. Select patients with TRK fusion-positive cancers may thus benefit from sequential TRK inhibitor therapy. TRK inhibition is well tolerated overall, particularly with first-generation agents, but occasional on-target adverse events such as dizziness, paraesthesias and weight gain can occur.

## Footnotes

* Note added in proof: The European Medicines Agency granted marketing authorisation for larotrectinib on 23 September 2019 as monotherapy for the treatment of adult and paediatric patients with solid tumours that display a neurotrophic tyrosine receptor kinase (*NTRK*) gene fusion, and who have disease that is locally advanced, metastatic or where surgical resection is likely to result in severe morbidity, and who have no satisfactory treatment options.
